# Short Report: Serological Evidence of Under-Reported Dengue Circulation in Sierra Leone

**DOI:** 10.1371/journal.pntd.0004613

**Published:** 2016-04-26

**Authors:** Jaime M. de Araújo Lobo, Christopher N. Mores, Daniel G. Bausch, Rebecca C. Christofferson

**Affiliations:** 1 Louisiana State University, School of Veterinary Medicine, Department of Pathobiological Sciences, Baton Rouge, Louisiana, United States of America; 2 Tulane University, New Orleans, Louisiana, United States of America; 3 Pandemic and Epidemic Diseases, World Health Organization, Geneva, Switzerland; Kenya Medical Research Institute, KENYA

## Abstract

Dengue virus (DENV) is thought to have emerged from a sylvatic cycle in Africa but has since become adapted to an urban-centric transmission cycle. These urban areas include villages in West Africa where DENV is not often routinely considered for patients presenting with febrile illnesses, as other endemic diseases (malaria, Lassa fever, e.g.) present with similar non-specific symptoms. Thus, dengue is likely under diagnosed in the region. These plaque reduction neutralization test-50 (PRNT50) screening results of patients presenting with fevers of unknown origin (FUO) at a clinic in Kenema, Sierra Leone indicate that all four serotypes of DENV likely circulate in areas surrounding Kenema. Using a more conservative PRNT80 cut-off value, our results still indicate the presence of antibody to all four serotypes in the region. Identifying alternate etiologies of FUOs in this region will assist clinicians in plan-of-care decisions as well as follow-up priorities. This is particularly relevant given the Ebola outbreak in the region, where diagnosis has a range of downstream effects ranging from correct allocation of medical resources, appropriate isolation of patients, and ultimately, a better informed public health sector.

## Author Summary

Identifying the infectious diseases in developing nations could assist in clinical response, disaster response, and in assessing the need for specific public health infrastructure and therapeutics. Here we tested serum from patients in Sierra Leone who sought treatment for fever (and which remained undiagnosed) for immunological reaction to dengue viruses 1–4. We used a plaque reduction neutralization test, where patient antibodies will neutralize virus and reduce the number of plaques formed by virus. We report both the typical 50% reduction as well as a more stringent 80% reduction in our cohort. We show that over 77% of patients in this study had moderate reaction (PRNT50) to at least one dengue virus type and many patients reacted to more than one dengue virus. We conclude that dengue is circulating in the region and may be a thus far undiagnosed etiology of fevers of unknown origin in the region.

## Introduction

Over the last few decades, there has been a worldwide re-emergence of arthropod-borne viral pathogens (arboviruses), particularly those transmitted by mosquitoes of which many are of the genus Flavivirus [1–4]. Despite the public health importance, the geographic range of the pathogens and their relative impact, the epidemiological characteristics linked to the arbovirus infection are poorly defined in many regions of the world, particularly in West Africa.

Due to the overlapping symptomology of dengue (DENV) and other endemic diseases (malaria, Lassa fever, e.g.), dengue is likely under diagnosed in the region[5]. To explore the potential of DENV to be an etiological agent of fevers of unknown origin in Sierra Leone, we performed serological testing of blood samples using the plaque reduction neutralization (PRNT) assay which assesses the serum neutralization capability to a viral pathogen [6, 7].

## Materials and Methods

### Ethics Statement

The enrollment of patients at the Kenema District hospital in Sierra Leone in the study and collection of blood was conducted under the study approved by the Tulane University Internal Review Board (IRB) and the Ethics Committee of Sierra Leone Ministry of Health, as well as the Louisiana State University IRB. After meeting study criteria and obtaining consent, blood samples were collected from patients in an acute stage of disease and subsequently on days 7 (late acute) and 28 (convalescence), when possible. Adults and parents/guardians of children provided written informed consent for inclusion in the study.

### Plaque Reduction Neutralization (PRNT) Assays

Samples were collected, inactivated, and stored as in [8, 9]. Additionally, patients included in this study were determined not to have malaria or Lassa fever also as in [8, 9]. Serum was first tested for the ability to neutralize representative strains of dengue (DENV) by PRNT to all four serotypes (DENV1-4, Table 1) as in [10], with the exception that we used BA1 diluent to dilute serum samples [11]. Prior to utilization in PRNT assays, concentrations of viral stocks were determined by plaque assay to determine the necessary dilution of stock virus yielding 100 plaque-forming units per 50 μl. To visualize neutralization via PRNT, patient samples were diluted 1:10 with BA1 diluent and screened for the ability to neutralize flaviviruses., using a 100 μl of virus-serum in a 1:1 mixture.

**Table 1:** Representative viruses used for PRNT and endpoint titration assays. Titers indicated were calculated based on a plaque assay.

Virus	Serotype	Strain	Origin	Titer (pfu/mL)
DENV	1	West Pacific74	Human, Naruu, 1974	1.1 * 10^6^
	2	16803	Human, Thailand,	2.5 * 10^7^/mL
	3	CH5548904500	Human, Thailand, 1973	9 * 10^3^
	4	LN 634441	Human, Malaysia 1988	7.41 * 10^6^

PRNTs were interpreted as follows: Three positive control wells were made per virus. Plaque reduction by patient antibodies to virus was expressed as the proportion of plaques formed in the serum/virus samples divided by the average number of plaques in the positive controls. Percent reduction was calculated as 1 minus the plaque reduction times 100. Reduction percentage values equal to or greater than 50% (PRNT50) were considered to have a positive result according to standard methods; those with values of 80% (PRNT80) were considered highly neutralizing [12].

## Results

**Fig 1:**
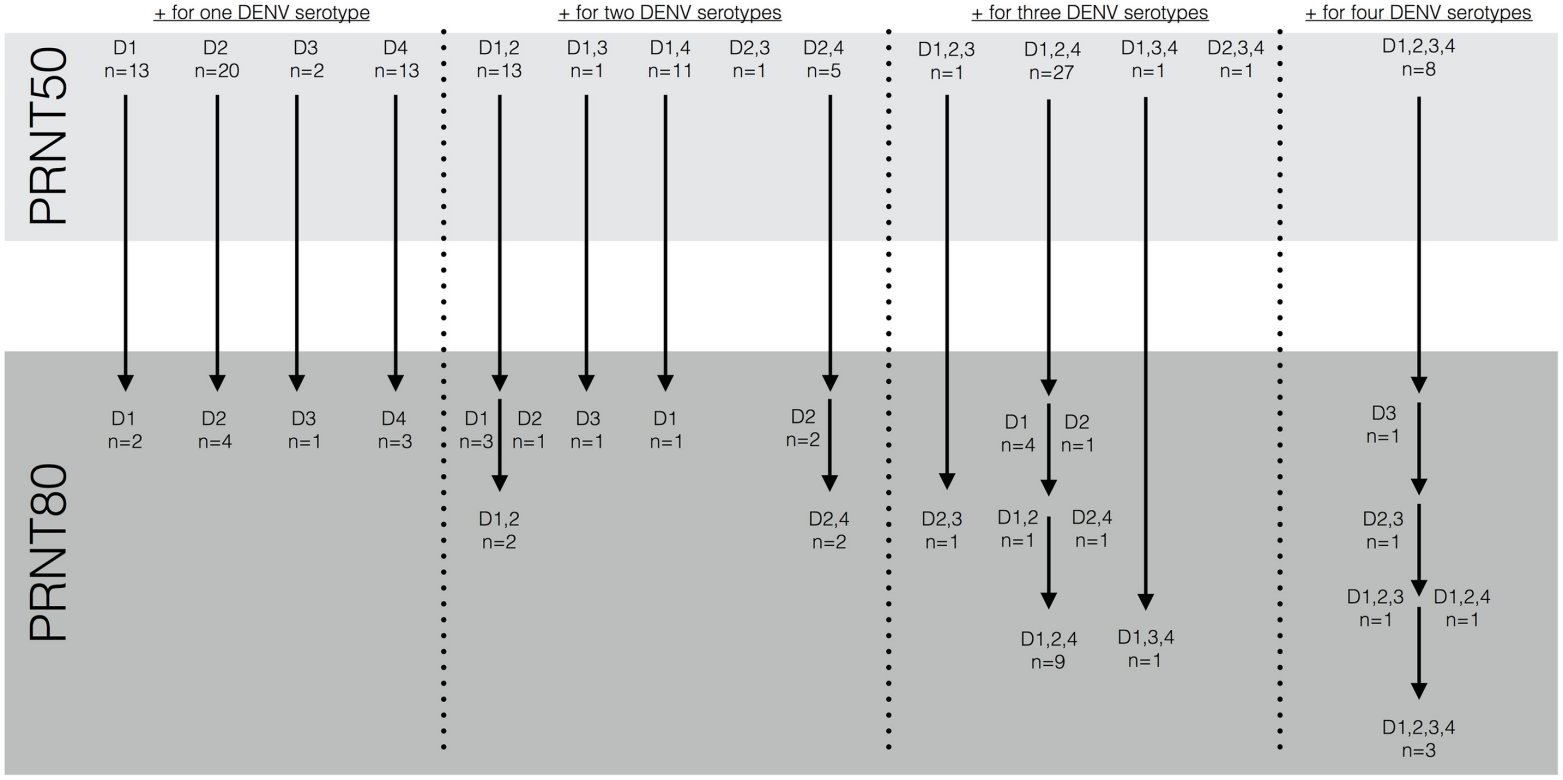
117 of 149 patients were positive for 1, 2, 3 or 4 serotypes of DENV as assessed by the PRNT50; patient serum was then further assessed for neutralizing capabilities at the PRNT80 level, often resulting in a lower order combination neutralization or no neutralization at all.

We tested 149 human serum samples from Sierra Leone for reaction to DENV, of which 32 were negative. The remaining 117 (78.52%) showed reduction at the PRNT50 level to at least one serotype, and there was evidence of exposure to all four serotypes. Fig 1 shows the breakdown of reactivity at both the PRNT50 level: 48 patients reacted to only 1 serotype, 31 reacted to two serotypes, 30 reacted to three serotypes, and 8 reacted to all four DENV serotypes. Ten of the 48 patients that reacted to a single serotype at the PRNT50 level showed neutralization at the PRNT80 level, as well. Of those patients reacting to two serotypes at the PRNT50 level, only four showed two-serotype neutralization at the PRNT80 level while another eight showed neutralization for a single serotype. Similarly, only 10 of the 30 patients reacting to three serotypes at the PRNT50 level showed neutralization to three serotypes at the PRNT80 level; 3 showed neutralization to two serotypes and five had neutralization to a single serotype. Finally, of the eight patients reacting to all four DENV serotypes at the PRNT50 level, only three showed neutralization capacity to all four at the PRNT80 level; two neutralized to three serotypes, one to two serotypes, and another to a single serotype (Fig 1). Full data and results provided in S1 Table.

## Discussion

Dengue has circulated in West Africa and disease manifestations have been reported [13–17]. But often, patients present with late stages of febrile illness and, since serological diagnostics are rarely administered in this region, malaria, typhoid fever, and other more common febrile etiologies are likely over-diagnosed [15]. Indeed as is the case of patients presenting to the Kenema District hospital in Sierra Leone, the first tests run are for malaria and Lassa fever [8, 9]. When these agents are ruled out, patients are given a diagnosis of “fever of unknown origin” (FUO). While our results cannot definitively indicate that DENV is the etiological agent of their current FUO, we show that there is a relatively high proportion of individuals that have been exposed to DENV. Thus, DENV should be considered a potential agent of some of these FUO.

Screening of patient serum indicates that all four serotypes of DENV likely circulate in West Africa, specifically in areas surrounding Kenema, Sierra Leone. As DENV is not often included in the battery of tests for FUO, it is probably under diagnosed. Utilizing a more stringent PRNT80 level, we have confidence that these patients are reacting to DENV; thus, at least some portion of the neutralizing antibodies are specific to DENV and not due to cross-reactivity to closely related Yellow Fever virus (for which a good portion of the population has been vaccinated or naturally exposed) [18, 19].

Our study is not without limitations. Power outages at our study site posed a challenge to maintaining frozen sera and proper storage of specimens and reagents. This potential obstacle could have resulted in the deterioration of antibodies, which raises the possibility that even our report underestimates the frequency of infection in patients [15]. The PRNT does not differentiate IgM from IgG antibody, and therefore these results cannot reliably inform us as the to incidence of the detected exposure events, including whether the febrile episodes that resulted in these patients presenting to the hospital are a result of those exposures.

The speculation that FUO are indeed due to DENV infections needs further study. However, our results do suggest that all four serotypes of DENV are clinically relevant and being transmitting in this region. Correct diagnosis and screening of patients is particularly important given the unprecedented outbreak of Ebola virus in the region [20]. Identifying which pathogens that are circulating and the potential alternate etiologies of the generic and often broad range of symptoms attributed to these more epidemic prone viruses (Ebola, Lassa, Marburg, e.g.) is critical not only for response and logistical efforts, but for correct allocation of medical resources, appropriate isolation of patients, and ultimately, a better informed public health sector.

## Supporting Information

S1 Table Shows the dengue serotype(s) each patient neutralized at the PRNT50 and PRNT80 levels at a 1:10 dilution of serum. (XLSX)
